# Host-pathogen interactions during early stages of bovine mastitis: divergent macrophage responses to distinct bovine-associated Staphylococci species and strains

**DOI:** 10.1007/s42770-026-01888-x

**Published:** 2026-02-22

**Authors:** Sarah Antonieta de Oliveira Veríssimo, Ygor Fagundes Ruas, Filipe Aguera Pinheiro, Luiza Campos Reis, Bernardina Amorim Uscata, Hiro Goto, Soraia Araújo Diniz, Adriana Cortez, Sarne De Vliegher, Marcos Bryan Heinemann, Eduardo Milton Ramos-Sanchez, Fernando Nogueira de Souza, Mônica Maria Oliveira Pinho Cerqueira

**Affiliations:** 1 Departamento de Tecnologia e Inspeção de Produtos de Origem Animal, Escola de Veterinária, Universidade Federal de Minas Gerais, Belo Horizonte, 31270-901 Brazil; 2 Centro Nacional de Pesquisa em Energia e Materiais (CNPEM), Ilum Escola de Ciência, Rua Lauro Vannucci, São Paulo, Campinas 1020, 13087-548 Brazil; 3 Laboratório de Soroepidemiologia e Imunobiologia, Faculdade de Medicina, Instituto de Medicina Tropical, Universidade de São Paulo, Av. Dr. Enéas de Carvalho de Aguiar 470, São Paulo, 05403-000 Brazil; 4 Departamento de Medicina Veterinária Preventiva e Saúde Animal, Faculdade de Medicina Veterinária e Zootecnia, Universidade de São Paulo, São Paulo, 05508-270 Brazil; 5 Escuela Profesional de Medicina Humana, Facultad de Medicina, Universidad Nacional Toribio Rodríguez de Mendoza de Amazonas, Chachapoyas, 01000 Peru; 6 Departamento de Salud Publica, Facultad de Ciencias de La Salud, Universidad Nacional Toribio Rodriguez de Mendoza de Amazonas, Chachapoyas, 01000 Peru; 7 Laboratório de Sorologia e Imunobiologia, Faculdade de Medicina, Instituto de Medicina Tropical, Universidade de São Paulo, São Paulo, 05403-000 Brazil; 8 Universidade Federal do Vale do Jequitinhonha e Mucuri – Campus Unaí, Unaí, 38610-000 Brazil; 9 Universidade Santo Amaro, UNISA, São Paulo, São Paulo, 04743-030 Brazil; 10 M-team and Mastitis and Milk Quality Research Unit, Department of Internal Medicine, Reproduction and Population Health, Faculty of Veterinary Medicine, Ghent University, Salisburylaan 133, Merelbeke, 9820 Belgium; 11 Veterinary Clinical Immunology Research Group, Departamento de Clínica Médica, Faculdade de Medicina Veterinária e Zootecnia, Universidade de São Paulo, São Paulo, 05508-270 Brazil

**Keywords:** Staphylococcus aureus, Non*-aureus* staphylococci, Mastitis, Immune response, Dairy cow

## Abstract

**Background:**

Bovine mastitis is the most economically significantdisease in dairy farming, with Staphylococcus aureus and Staphylococcus chromogenes asprominent agents, the former posing a major threat. As principal immune sentinels in themammary gland, macrophages orchestrate early pathogen recognition and immune activation,critically influencing the trajectory and outcome of infection. Thus, this study aimed tocharacterize the early macrophage responses to distinct bovine-associated S. aureus and S.chromogenes strains.

**Methods:**

Here, RAW 264.7 cells were challenged with four differentstrains: S. aureus [isolated from nose (SN), and intramammary infection (IMI)] and S.chromogenes [IMI, and teat apex (TA)] were evaluated after 90- and 180-min. Nitric oxide (NO)production was analyzed in the supernatants, and mRNA levels of IL-1β, IL-18, NLRP3, NOS2,Arg1, Bax, and Bcl2 were assessed in the cells.

**Results:**

Macrophages challenged with S. aureusIMI strains showed elevated Nos2 expression but negligible NO production, indicating a potentialimmune evasion mechanism. The commensal S. aureus SN strain uniquely maintained arginaseexpression, suggesting M2-like polarization that may promote immune tolerance and bacterialcolonization. Both S. aureus strains significantly upregulated the anti-apoptotic Bcl2 gene, atranscriptional response that may be associated with host cell survival, which may facilitatebacterial intracellular persistence. In contrast, S. chromogenes strains induced strong NOS2expression, robust NLRP3 inflammasome activation, and increased IL-1β production, indicatingM1 polarization and a pro-inflammatory response. The pro-apoptotic Bax gene showed an earlydecrease followed by a later increase exclusively in S. aureus-infected macrophages, indicating atime-dependent transcriptional modulation of apoptosis-related genes.

**Conclusions:**

Thesegenotype-dependent macrophage responses reveal complex immune modulation shaping mastitis pathogenesis. However, our findings are based solely on transcriptional data on the murine cells and require further validation.

## 1. Introduction

Bovine mastitis is one of the most economically impactful diseases in dairy farming worldwide, with annual losses estimated at US$27 billion per year [1], and it is still responsible for approximately 60% of antimicrobial use on average dairy farms [2]. Furthermore, bovine mastitis negatively impacts public health, the dairy industry’s image, and animal welfare [2]. Alongside ongoing efforts to reduce antibiotic use in response to antimicrobial resistance, exploring nonantibiotic alternatives requires understanding host-pathogen interactions, which is essential for developing strategies to maintain mammary gland health without antimicrobials [3–5].

Among bovine mastitis pathogens, *Staphylococcus aureus* is a major mastitis pathogen that poses the most significant challenge because of its pathogenicity and sophisticated mechanisms of immune evasion, which hinder efforts to control and eliminate it [5]. Furthermore, *S. aureus* is frequently isolated from persistent intramammary infections (IMI) and extramammary sites [6, 7]. While *S. aureus* remains the primary concern, non-*aureus* staphylococci (NAS) comprise a diverse group of species that, although usually regarded as minor mastitis pathogens, are the most commonly isolated organisms from aseptically collected bovine milk samples worldwide [8, 9]. Among them, *S. chromogenes* is the most prevalent species. Although it is typically regarded as a minor mastitis pathogen or even as a commensal, *S. chromogenes* can still cause persistent IMIs [8, 9]. Notably, some *S. chromogenes* strains exert in vitro and in vivo protective effects against major mastitis pathogens, such as *S. aureus*, *Streptococcus uberis*, and *Escherichia coli*, suggesting a potential beneficial role in the mammary gland health [5, 10–14]. Although *S. aureus* and *S. chromogenes* are both prevalent, their differences in severity and the extent of the infection are remarkable. Therefore, understanding these differences in the infectious process may provide clues for prevention and control.

Macrophages are the predominant milk leukocytes in healthy bovine mammary glands [15]. In addition to being present in the milk, they can be interspersed among mammary tissue as ductal macrophages [16]. Macrophages play multifaceted roles, essential for initiating, sustaining, and resolving inflammation, serving as the first immune cells to detect and respond to invading pathogens in the mammary gland [17, 18]. Therefore, upon bacterial invasion, macrophages recognize pathogen-associated molecular patterns (PAMPs) through pattern recognition receptors (PRRs), triggering a signaling cascade that results in the release of proinflammatory cytokines and chemokines, which further exacerbate local inflammation and facilitate the recruitment of additional immune cells to the infection site [19].

A subset of PRRs in the cytosol plays a key role in activating inflammasomes and inflammatory caspases. Among the inflammasomes, NOD-like receptor family, pyrin domain containing 3 (NLRP3) is the most studied and characterized. Caspases cleave the proinflammatory cytokines interleukin (IL)-1β and IL-18, converting them into their mature forms. The mature cytokines then trigger pyroptotic cell death, a form of inflammatory cell death that enhances the immune response. These cytokines are critical for immunity against infections, as they coordinate the activation and recruitment of immune cells, induce the production of additional inflammatory molecules, and regulate antimicrobial mediators, such as nitric oxide (NO), in macrophages [20]. Nitric oxide is synthesized from the amino acid arginine by nitric oxide synthase (NOS). One of the key isoforms of NOS, NOS2, also known as inducible nitric oxide synthase, is critical for producing NO in response to inflammatory stimuli, contributing to the antimicrobial activity of macrophages. Interestingly, NO also regulates inflammasome activity in an autocrine and paracrine manner, influencing both the local inflammatory environment and the broader immune response [21]. In this context, host macrophages can be classified into two main subsets based on the expression of two specific enzymes, *NOS2* and arginase 1 (*ARG1*), respectively. M1 macrophages, classically activated, predominantly express *NOS2* and are involved in pathogen clearance by producing inflammatory mediators like NO, and can release high levels of pro-inflammatory cytokines. In contrast, M2 macrophages, which are alternatively activated, express *ARG1* and produce high levels of anti-inflammatory cytokines. ARG1 metabolizes arginine into ornithine and urea, which is crucial in resolving inflammation, promoting tissue repair, and regulating NO production. This dichotomy between *NOS2* and *ARG1* expression highlights the opposing functions of these macrophage subsets: M1 macrophages drive pro-inflammatory responses, while M2 macrophages facilitate tissue repair and immune resolution. In this regard, the ability of some pathogens to manipulate the expression of *ARG1* and *NOS2* is a crucial strategy for immune evasion and enhancing their survival [22]. Therefore, understanding the mechanisms underlying macrophage polarization, inflammasome activation, and cell death induction is crucial for deciphering how different pathogens modulate the host immune response. While inflammasome activation and macrophage polarization have been addressed in previous studies on bovine mastitis [20, 23], the strain-specific effects of *Staphylococcus aureus* and *Staphylococcus chromogenes*, whether udder-adapted or isolated from extramammary sites, on macrophage function remain largely unexplored.

The diversity of bovine-associated staphylococcal species and strains within species reveals substantial variations in their ability to induce inflammation, as determined by somatic cell counts in milk, persistence in IMI, antimicrobial resistance, pathogenicity, and epidemiological and ecological behavior [5, 8, 24–28]. Thus, it is reasonable to assume that distinct staphylococcal strains isolated from persistent infections or extramammary niches also elicit distinct macrophage responses. Therefore, the present study investigates the early innate immune responses of macrophages to bovine-associated *S. aureus* and *S. chromogenes* strains, by assessing nitric oxide production and the expression of key inflammatory, regulatory, and apoptotic genes to identify strain-specific immune modulation patterns. Therefore, the data from the present study raise further concerns about how persistent or commensal staphylococcal infections prime the host cell response and may contribute to the control or progression of an upcoming infection.

## Materials and methods

### 1.1. Staphylococcal strains and growth conditions

Four well-studied distinct staphylococcal strains comprising two diverse *Staphylococcus aureus* strains and two *Staphylococcus chromogenes* strains were used in this study [5, 6, 11–14, 25–29]. The first *S. aureus* strain (spa type t605) was isolated from a persistent subclinical intramammary infection (IMI) [5, 6, 27, 29], whereas the other strain was isolated from a nasal swab (NS; spa type t098) [5, 6, 27, 29]. Two dissimilar *S. chromogenes* strains were used: the first *S. chromogenes* strain was isolated from a heifer’s teat apex (TA) [5, 11, 12, 24–29], whereas the second strain was isolated from a persistent IMI ( IMI [5, 11–14, 24–29]; multilocus sequence type 1, “Chromo-MAS” in Huebner et al., 2021 [30]). All strains were stored at -80 °C and thawed at room temperature. Following thawing, the samples were cultured on 5% sheep blood agar plates. Subsequently, fresh colonies of each bacterium were cultivated for 12 to 16 h in brain heart infusion broth (BHI) at 37 °C. All the samples were diluted 1:1000 and incubated in BHI broth until they reached their respective late exponential growth phases, corresponding to an optical density at 600 nm (OD600) of 0.6–0.8 using a spectrophotometer. After bacterial growth, the bacterial suspension was centrifuged at 2500 × g for 15 min at 4 °C, and washed twice with sterile Dulbecco’s phosphate-buffered saline (DPBS) solution without calcium chloride and magnesium chloride (cat. no. 14190185, Gibco, Paisley, United Kingdom). Afterward, the bacteria were resuspended in antibiotic-free RPMI-1640 cell culture medium (cat. no. R7638, Sigma Aldrich, St. Louis, USA). The live bacteria count was determined using a 10-fold serial dilution, in which 10 µL of each staphylococci strain was cultured on BHI agar until live bacteria were reliably counted to determine the number of colony-forming units (CFU) [26]. The inoculum was adjusted to 5 × 10^6^ CFU/mL and stored at -80 °C for further processing for a maximum of 7 days.

### 1.2. Macrophage cell line culture

When this study was conducted, no commercially available bovine macrophage line was available; therefore, we used the RAW 264.7 murine macrophage cell line to minimize host variability. This macrophage cell line exhibits consistent growth characteristics and has been extensively used in several studies, including those related to bovine mastitis [29, 31–34]. The macrophages were cultured for 4 days in RPMI-1640 medium supplemented with 10% heat-inactivated fetal bovine serum (FBS; cat. no. F9665; Sigma Aldrich) and 1% antibiotic-antimycotic solution (cat. no. 15240-062; Life Technologies) at 37 °C with 5% CO_2_, as previously described [29]. Cell viability was assessed using a trypan blue exclusion test. The cell concentration was adjusted according to the specific needs of each experimental setup.

### 1.3. Macrophage challenge with distinct Staphylococcal strains

The RAW 264.7 murine macrophage cells were distributed across 24-well plates and incubated in a humidified chamber with 5% CO_2_ at 37 °C for 1 h to promote cell adhesion. Following adhesion, the cells were washed with RPMI-1640 cell culture medium (cat. no. R7638, Sigma Aldrich, St. Louis, USA) to remove any remnants of the antibiotic-antimycotic mixture, preventing bacterial death. A multiplicity of infection (MOI) of 1 was used in the infection experiments to more accurately mimic the pathogenesis of a natural infection during its early stages [29, 35], with two incubation times (90 min and 180 min). Briefly, 5 × 10^5^ cells were plated per well in 500 µL of antibiotic-free RPMI-1640 with 5% FBS with 100 µL of the bacterial inoculum (5 × 10^5^ staphylococci). Macrophages were analyzed in technical triplicate per plate for each condition, and the experiment was independently repeated twice, resulting in six samples per bacterial strain per time point. After the incubation, the supernatant was collected for NO quantification. At the same time, the cells were collected and stored in 1 mL of TRIzol (cat. A33248, Thermo Fisher Scientific, Waltham, Massachusetts, USA) at -80 °C.

### 1.4. mRNA quantification of *IL-1β*, *IL-18*, *NLRP3*, *Nos2*, *Arg1*, *Bax* and *Bcl2*

#### 1.4.1. RNA purification

Total RNA was extracted from 5 × 10^5^ cells/mL resuspended in 1 mL of TRIzol (cat. A33248, Thermo Fisher Scientific, Waltham, Massachusetts, USA), followed by the addition of 200 µL of chloroform. Centrifugation was performed at 13,000 × g for 15 min at 4 °C. The separated aqueous phase was mixed with isopropanol (v/v), and the mixture was incubated for 30 min at room temperature. Subsequently, centrifugation resulted in a cellular pellet to which 1 mL of 75% ethanol was added. After another centrifugation, the supernatant was discarded. The pellet was left at room temperature for 10 min (until the ethanol evaporated) and then suspended in RNase-free water. The obtained RNA was quantified and analyzed by spectrophotometry at 260/280 nm and 260/230 nm using a NanoDrop spectrophotometer (Thermo Fisher Scientific, Waltham, Massachusetts, USA).

#### 1.4.2. DNA synthesis

For cDNA synthesis, 1 µg of total RNA sample was combined with 6 µL of the High-Capacity cDNA Reverse Transcription Kit reagent (Applied Biosystems, Foster City, California, USA), and cDNA was synthesized following the manufacturer’s recommendations.

#### 1.4.3. Real-time polymerase chain reaction (qPCR)

For the qPCR analysis, a panel of 8 genes was selected, including *Arg1*, *Nos2*,* IL-18*, *IL-1β*,* NLRP3*, *Bax*, *Bcl2*, and *β-actin*. The primer sequences employed for amplification are detailed in Table 1. For the qPCR, 1 µL of cDNA was combined with 12.5 µL of SYBR^®^ Green reagent (2X) (Applied Biosystems, USA), 1 µL of each primer at 5.0 pMol/µL, and 9.5 µL of autoclaved Milli-Q water following the manufacturer’s instructions. The relative quantification of mRNA expression involved comparing the threshold cycle (CT) of the target genes (*IL-18*, *IL-1β*, *NLRP3*, *Nos2*, *Arg1*, *Bax*, and *Bcl2*) with the constitutive *β-actin* gene. The results were normalized for expression analysis by subtracting the CT obtained for the constitutive gene from the CT of the target gene for each sample (ΔCT). For comparisons between different groups, calculations were subsequently performed relative to the ΔCT value of uninfected and unstimulated cells only (ΔΔCT), and the final value was obtained using the following equation: 2⁻ΔΔCT [36].

**Table 1:** Primer sequences used for qPCR amplification of *Arg1*, *Nos2*, *IL-18*, *IL-1β*, *NLRP3*, *Bax*, *Bcl2*, and *β-actin* and their respective product size

Gene	Forward Primer (5′ → 3′)	Reverse Primer (5′ → 3′)		Annealing Temperature (°C)	Amplicon Size (pb)	
***Arg1***	AGC ACT GAG GAA AGC TGG TC	CAG ACC GTG GGT TCT TCA CA		60	136	
***Nos2***	AGA GCC ACA GTC CTC TTT GC	GCT CCT CTT CCA AGG TGC TT		60	138	
***IL-18***	CCT GAA GAA AAT GGA GAC CTG GAA	ACA CAG GCT GTC TTT TGT CAA CGA		60	115	
***IL-1β***	TCA TCT TTG AAG AAG AGC CCA TCC	CGG AGC CTG TAG TGC AGT TGT CTA		60	100	
***NLRP3***	TAG ACA ACT GCA GCC TCA CCT CAC	ATTTCACCCAACTGTAGGCTCTGC		60	181	
***Bax***	AAC TGG ACA GTA ACA TAG AG	TTG CTG GCA AAG TAG AAA AG		60	148	
***Bcl2***	GAT TGT GGC CTT CTT TGA G	GTT CCA CAA AGG CAT CC		60	138	
***β-actin***	GCC TTC CTT CTT GGG TAT GGA ATC	ACG GAT GTC AAC GTC ACA CTT CAT		60	81	

### 1.5. Nitric oxide quantification

Nitrite (NO₂⁻), a stable degradation product of nitric oxide released by the cells, was quantified in cell culture supernatants using the Griess reaction [37], which specifically detects nitrite and does not measure nitrate (NO₃⁻). Following treatment periods (90 min and 180 min) with each staphylococcal infection, the supernatant from each well was collected for the Griess reaction. For NO quantification, 50 µL of culture supernatant and 50 µL of Griess reagent (Solution A: 1% sulphanilamide in distilled water and Solution B: 0.1% N-(1-naphthyl) ethylenediamine dihydrochloride and 2.5% phosphoric acid in distilled water) (v/v) were added to each well of a 96-well plate. The mixture was incubated for 10 min at room temperature in the dark. The nitrite concentration was determined based on a standard curve established from known concentrations of NaNO_2_. Analysis was performed using a plate spectrophotometer (Thermo Fisher Scientific, Waltham, Massachusetts, USA) with a 540 nm filter.

### 1.6. Statistical analysis

Before the analyses, the normality and homoscedasticity of the variables were assessed using the Shapiro-Wilk and Bartlett tests, respectively. The variables related to qPCR and nitric oxide production exhibited a nonparametric distribution, and normalization through transformation was not feasible due to high coefficients of variation. The Kruskal-Wallis test was applied to assess differences among groups for the qPCR variables, followed by Dunn’s post hoc test with Holm correction to adjust for multiple comparisons. For paired comparisons across different time points, the Wilcoxon signed-rank test was used. All analysis considered a significance level (α) of 0.05. Comparisons were conducted at two levels: Level 1 involved individual examination of the time and bacteria effects, whereas Level 2 explored the interaction between bacteria and time effects. The statistical software used was R version 4.3.1.

## 2. Results

### 2.1. Relative expression of NOS2 and arginase genes and NO production

**Fig. 1:**
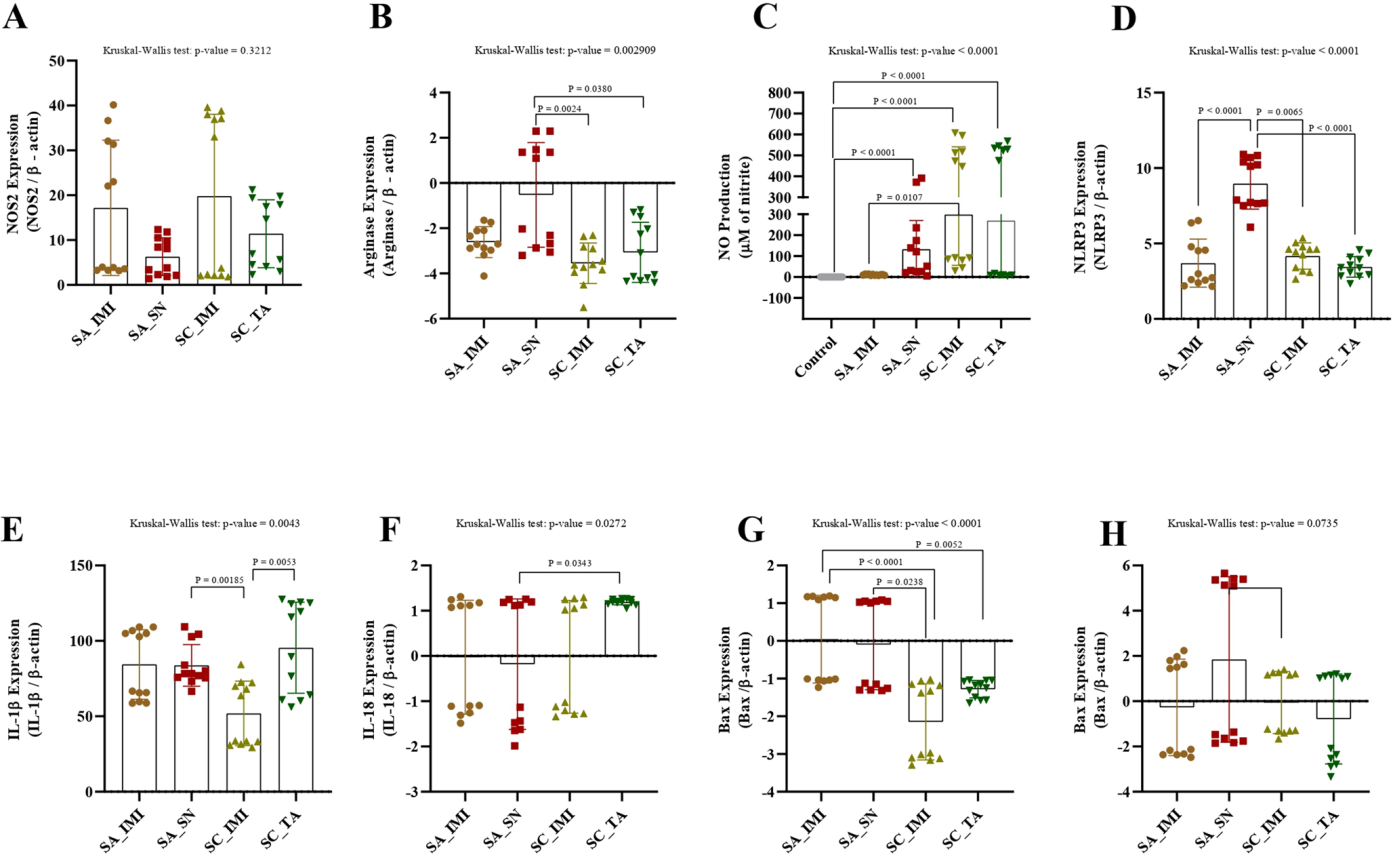
**RAW 264.7 cells’ responses to distinct staphylococcal strains after infection.** (**A**) Relative expression of *NOS2*, normalized to ***β-actin*** and expressed as fold change relative to unstimulated control cells. (**B**) Relative expression of arginase, normalized to ***β-actin*** and expressed as fold change relative to unstimulated control cells. (**C**) Nitric oxide (NO) production, measured as nitrite concentration (µM). (**D**) Relative expression of the inflammasome component *NLRP3*, normalized to ***β-actin*** and expressed as fold change relative to unstimulated control cells. (**E**) Relative expression of the pro-inflammatory cytokine *IL-1β*, normalized to ***β-actin*** and expressed as fold change relative to unstimulated control cells. (**F**) Relative expression of the cytokine *IL-18*, normalized to ***β-actin*** and expressed as fold change relative to unstimulated control cells. (**G**) Relative expression of the pro-apoptotic gene *Bax*, normalized to ***β-actin*** and expressed as fold change relative to unstimulated control cells. (**H**) Relative expression of the anti-apoptotic gene *Bcl2*, normalized to ***β-actin*** and expressed as fold change relative to unstimulated control cells. Legend: SA_IMI - *Staphylococcus aureus* isolated from a persistent intramammary infection; SA_SN - *Staphylococcus aureus* isolated from nasal swab; SC_IMI - *Staphylococcus chromogenes* isolated from a persistent intramammary infection; SC_TA - *Staphylococcus chromogenes* isolated from a teat apex. Bars represent the mean ± standard deviation

**Fig. 2:**
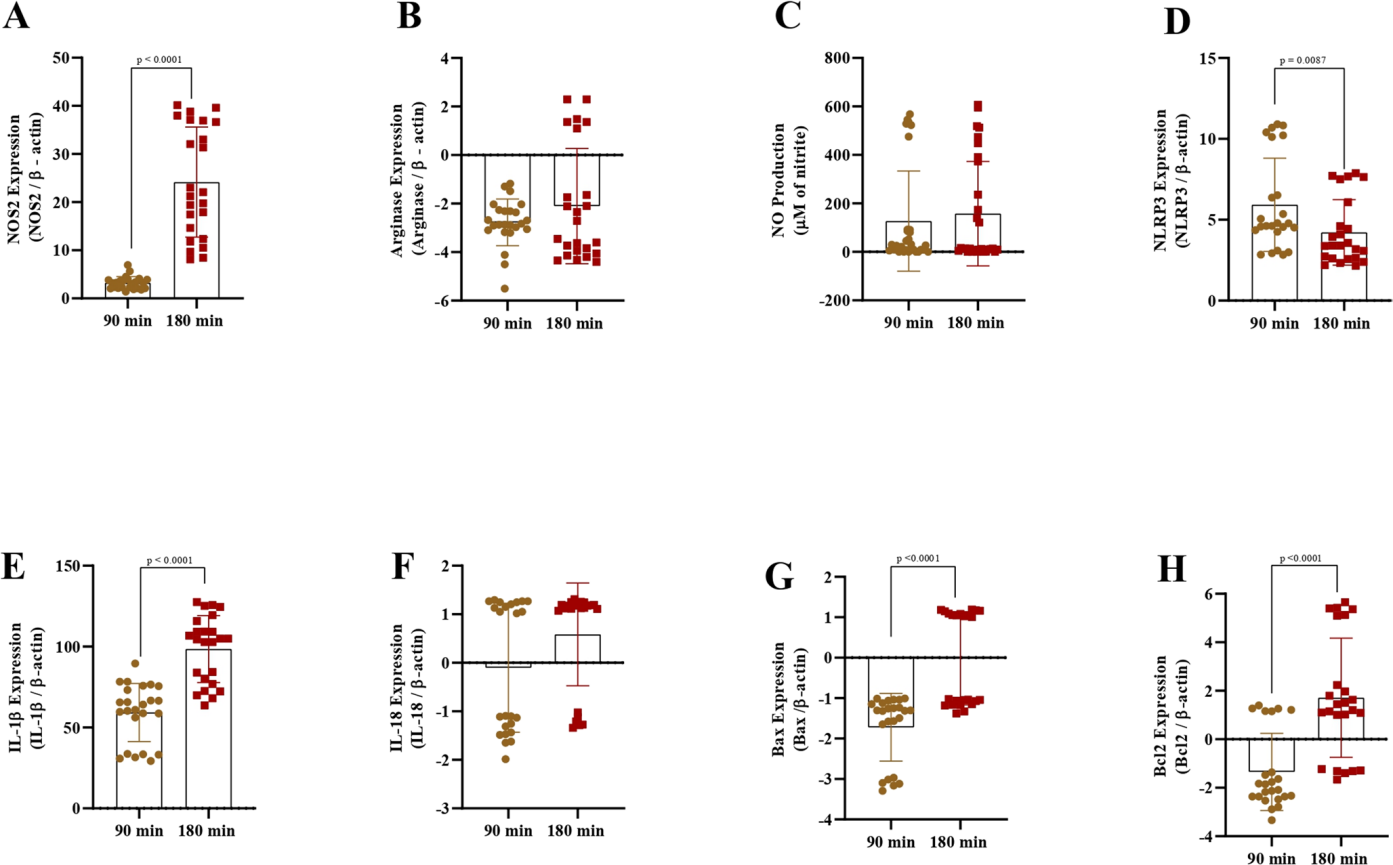
**Time-dependent RAW 264.7 cells’ responses to staphylococcal infections after 90 and 180 min of infection.** (**A**) Relative expression of *NOS2* normalized to *β-actin*. (**B**) Relative expression of arginase normalized to *β-actin*. (**C**) Nitric oxide (NO) production, measured as nitrite concentration (µM). (**D**) Relative expression of the inflammasome component *NLRP3* normalized to *β-actin*. (**E**) Relative expression of the pro-inflammatory cytokine *IL-1β* normalized to *β-actin*. (**F**) Relative expression of the cytokine *IL-18* normalized to *β-actin*. (**G**) Relative expression of the pro-apoptotic gene *Bax* normalized to *β-actin*. (**H**) Relative expression of the anti-apoptotic gene *Bcl2* normalized to *β-actin*. Bars represent the mean ± standard deviation

**Fig. 3:**
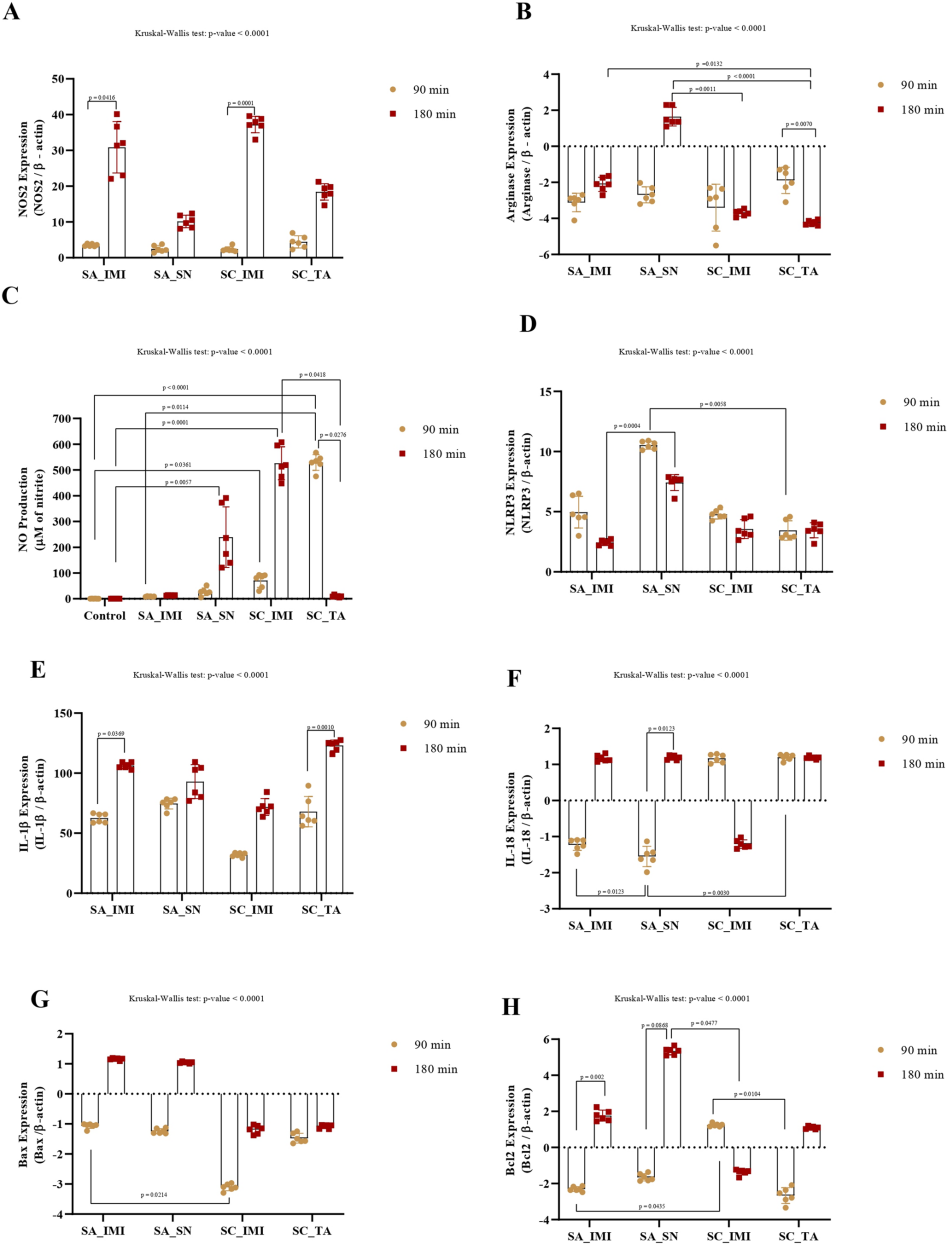
**Comparative analysis of RAW 264.7 cells responses to different staphylococcal strains at 90- and 180-minute post-infection.** (**A**) Relative expression of *NOS2*, normalized to ***β-actin*** and expressed as fold change relative to unstimulated control cells. (**B**) Relative expression of arginase, normalized to ***β-actin*** and expressed as fold change relative to unstimulated control cells. (**C**) Nitric oxide (NO) production, measured as nitrite concentration (µM). (**D**) Relative expression of the inflammasome component *NLRP3*, normalized to ***β-actin*** and expressed as fold change relative to unstimulated control cells. (**E**) Relative expression of the pro-inflammatory cytokine *IL-1β*, normalized to ***β-actin*** and expressed as fold change relative to unstimulated control cells. (**F**) Relative expression of the cytokine *IL-18*, normalized to ***β-actin*** and expressed as fold change relative to unstimulated control cells. (**G**) Relative expression of the pro-apoptotic gene *Bax*, normalized to ***β-actin*** and expressed as fold change relative to unstimulated control cells. (**H**) Relative expression of the anti-apoptotic gene *Bcl2*, normalized to ***β-actin*** and expressed as fold change relative to unstimulated control cells. Legend: SA_IMI - *Staphylococcus aureus* isolated from a persistent intramammary infection; SA_SN - *Staphylococcus aureus* isolated from nasal swab; SC_IMI - *Staphylococcus chromogenes* isolated from a persistent intramammary infection; SC_TA - *Staphylococcus chromogenes* isolated from a teat apex. Bars represent the mean ± standard deviation. Only significant comparisons between different time points within the same bacterial group, and between different bacterial groups at the same time point, are shown

In the present study, the relative expression of the *Nos2* gene did not significantly differ among different staphylococcal strains (*P* = 0.32; Fig. 1A). However, a greater relative expression of the *Nos2* gene was observed after 180 min of infection (*P* < 0.0001; Fig. 2A). Despite the similar behavior among distinct staphylococcal strains at the early stage of infection (90 min), significant variations among staphylococcal strains in the relative expression of the *Nos2* gene became evident after 180 min, with only *S. aureus* IMI and *S. chromogenes* IMI increasing significantly the *Nos2* gene expression (*P* < 0.0001; Fig. 3A). The time did not affect the relative expression of the *Arg1* gene (*P* = 0.94; Fig. 2B). Among the staphylococci strains, the relative expression of the *Arg1* gene was different between strains (*P =* 0.003), with *S. aureus* SN expressing higher expression than *S. chromogenes* IMI (*P =* 0.0024) and *S chromogenes* TA (*P =* 0.046; Fig. 1B). *S. aureus* SN was the unique isolate in which the relative expression of the *Arg1* gene was not inhibited in infected macrophages, particularly at 180 min (*P* < 0.0001; Fig. 3B). NO production by macrophages was not statistically different between 90 min and 180 min of infection than at 90 min (*P* = 0.82; Fig. 2C). However, when comparing the different bacterial strains, significant differences were observed. At both 90 and 180 min, macrophages infected with the *S. aureus* IMI strain did not exhibit increased nitric oxide production relative to controls (Fig. 3C). In contrast, *S. aureus* SN, *S. chromogenes* IMI, and *S. chromogenes* TA triggered a higher NO production by macrophages than the control (*P* < 0.0001; Fig. 1C). Distinct from the other staphylococcal strains, *S. chromogenes* TA was the only one that led to a drastic increase in NO production as early as 90 min, followed by a significant decrease thereafter (Fig. 3C).

### 2.2. Relative expression of the NLRP3, IL-1β, and IL-18 genes

The relative expression of the NLRP3 gene was significantly influenced by the bacterial strain (*P <* 0.0001; Fig. 1D), the incubation time (*P =* 0.0087; Fig. 2D), and when comparing different strains at different point times (*P <* 0.0001; Fig. 3D). Among the studied staphylococci, the *S. aureus* SN led to the highest relative expression of the *NLRP3* gene by macrophages (Fig. 1D), especially at 90 min (Fig. 3D), which is significantly higher than *S. chromogenes* TA (*P* = 0.006; Fig. 3D).

The relative expression of the *IL-1β gene* by macrophages was significantly influenced by the bacterial strain (*P <* 0.004; Fig. 1E), the incubation time (*P <* 0.0001; Fig. 2E), and when comparing different strains at different point times (*P <* 0.0001; Fig. 3E). After experimental infection with *S. chromogenes* IMI, the relative expression of the *IL-1β gene* by macrophages was significantly lower than *S. aureus* SN (*P =* 0.02) and *S. chromogenes* TA (*P =* 0.005; Fig. 1E) but was not significantly different from that induced by *S. aureus* IMI. Furthermore, macrophages’ relative expression of the *IL-1β* gene was higher at 180 min of infection than at 90 min (*P* < 0.001; Fig. 2E).

The relative expression of the *IL-18 gene* by macrophages was significantly influenced by the bacterial strain (*P <* 0.03; Fig. 1F) and when comparing different strains at different point times (*P <* 0.0001; Fig. 3F); however, it was not influenced by time (*P =* 0.15; Fig. 2F). Since all the mean values of the strains for IL-18 expression by macrophages varied only ± 1-fold compared with the control, these results are considered not biologically significant despite some observed significant differences (Figs. 1F and 3F).

### 2.3. Relative expression of the pro-apoptotic Bax and anti-apoptotic Bcl2 genes

The relative expression of the *Bax gene* by macrophages was significantly influenced by the bacterial strain (*P <* 0.0001; Fig. 1G), the incubation time (*P <* 0.0001; Fig. 2G), and when comparing different strains at different point times (*P <* 0.0001; Fig. 3G). Indeed, the relative expression of the *Bax* gene in macrophages was significantly downregulated at 90 min post-challenge compared to 180 min post-challenge (*P* < 0.0001; Fig. 2G). At 90 min, the relative expression of the pro-apoptotic *Bax* gene was significantly downregulated only when macrophages were infected with *S. chromogenes* IMI strain compared to *S. aureus* IMI (*P* = 0.02; Fig. 3G). Interestingly, while all staphylococcal strains inhibited the pro-apoptotic Bax gene after 90 min of infection, its expression started to increase after 180 min exclusively in macrophages infected with *S. aureus*, regardless of the strains; however, this increase was not significant (Fig. 3G).

The relative expression of the *Bcl2 gene* by macrophages was significantly influenced by incubation time (*P <* 0.0001; Fig. 2H) and when comparing different strains at different point times (*P <* 0.0001; Fig. 3H), however, when analysing only the bacterial strain, it was not significant (*P =* 0.07; Fig. 1H). The relative expression of the *Bcl2* gene by macrophages was greater 180 min after the experimental challenge than 90 min after the challenge (*P* < 0.001; Fig. 2H). Among all groups, *S. chromogenes* IMI induced a higher *Bcl2* expression by macrophages than *S. aureus* IMI (*P* = 0.04) and *S. chromogenes* TA (*P* = 0.01; Fig. 3H). Interestingly, both *S. aureus* strains induce higher expression of the *Bcl2 gene* by macrophages at 180 min than at 90 min, a pattern not observed with the *S. chromogenes* strains (Fig. 3H).

## 3. Discussion

The distinct impacts of *S. aureus* and *S. chromogenes* on udder health, milk production, and the severity of the inflammatory response highlight the need to understand their early interactions with the host immune system. This study focused on macrophages, pivotal cells in orchestrating the immune response, to unravel how these pathogens influence the initial stages of infection.

The functional polarization of macrophages is deeply intertwined with arginine metabolism, shaping their roles in health and disease. This polarization governs their antimicrobial and immunoregulatory functions and influences critical inflammatory pathways. The NLRP3 inflammasome represents a central mediator of inflammation in macrophages, whose activity can be indirectly modulated by nitric oxide produced via inducible nitric oxide synthase (*NOS2*). The elevated NO levels enhance pathogen clearance but can also contribute to inflammatory tissue damage and programmed cell death, such as pyroptosis. Conversely, M2 macrophages, characterized by arginase expression, counterbalance inflammation by metabolizing arginine into ornithine, promoting tissue repair and resolving inflammation. This dichotomy underscores the complex interplay between macrophage polarization, inflammasome regulation, and the outcomes of inflammation, cell death, and tissue homeostasis [38, 39].

In this context, our study demonstrates that both *S. chromogenes* strains induce robust *NOS2* expression while suppressing arginase activity, indicating an M1 macrophage polarization. Furthermore, these responses are accompanied by increased *NLRP3* mRNA expression and elevated transcription of pro-inflammatory cytokines, including *IL-1β* [38, 39]. Together, these findings suggest that *NLRP3* transcriptional upregulation may be associated with M1 polarization and contribute to shaping the inflammatory response. These findings align with our previous observations of elevated IL-1β concentrations in milk following mammary gland challenges with these *S. chromogenes* strains in lactating dairy heifers [25]. However, no substantial variations in *IL-18* expression were observed. This discrepancy may arise from inherent differences in the regulation of their precursors: pro-IL-1β is not expressed under basal conditions, while pro-IL-18 is constitutively present in macrophages, monocytes, dendritic cells, astrocytes, and microglia. Furthermore, a stronger priming signal may preferentially enhance pro-IL-1β levels, resulting in a cytokine secretion profile predominantly characterized by IL-1β rather than IL-18 [38].

Macrophages challenged with *S. aureus* IMI exhibited elevated *NOS2* expression but negligible NO production, highlighting the ’pathogen’s potential immune evasion strategy. These findings may be related to MicroRNAs such as miR-125a-5p and miR-301a are likely involved in regulating *NOS2* mRNA and protein levels, potentially by modulating key signalling pathways or transcription factors that control *NOS2* expression, a mechanism that remains to be fully elucidated [40]. This dysregulation of NO production may contribute to the pathogen’s persistence in intramammary infection. Conversely, macrophages challenged with *S. aureus* SN, a commensal strain, showed no suppression of arginase (*Arg1*) mRNA expression, a transcriptional profile often associated with a shift toward M2-like responses. Nonetheless, it is important to acknowledge that transcriptomic markers alone provide limited evidence of functional polarization. Although arginase is a hallmark of IL-4- and IL-13-stimulated macrophages with anti-inflammatory functions, it can also be co-expressed with *NOS2* under certain conditions, reflecting the plasticity of macrophage responses [41]. These findings suggest that commensal bacteria like *S. aureus* SN may help to maintain immunological tolerance [42]. Therefore, we hypothesize that this transcriptional response may be associated with colonization by suppressing pro-inflammatory cytokine expression and potentially inhibiting the recruitment of immune cells, which could favor pathogen establishment in the host. These results underscore genotype-dependent host transcriptional responses to *S. aureus* and highlight the complexity of host-pathogen interactions, which may vary according to bacterial genotype, as supported by previous studies [43, 44]. Furthermore, both *S. aureus* strains were associated with increased *NLRP3* mRNA expression in macrophages, accompanied by an abundant *IL-1β* gene expression but not *IL-18*. Consistent with our findings, primary microglia challenged with *S. aureus* exhibited elevated IL-1β production, while IL-18 release remained relatively subdued [45].

Furthermore, an upregulation of *Bcl2* gene was observed at 180 min in both *S. aureus* strains, in clear contrast to *S. chromogenes* strains. This pattern may reflect the ability of *S. aureus* to persist intracellularly within macrophages, providing a refuge that supports their survival and spread [46, 47]. Similarly, our results are consistent with transcriptome analyses of human monocyte-derived macrophages, where *S. aureus* infection triggers the anti-apoptotic machinery, upregulating anti-apoptotic genes while downregulating pro-apoptotic determinants [48].

Taken together, these findings provide mechanistic insights into strain-specific macrophage transcriptional responses and may help inform future studies exploring immunomodulatory strategies in the context of bovine mastitis. The distinct pro-inflammatory transcriptional profile induced by *S. chromogenes* suggests a differential engagement of macrophage responses that warrants further investigation, particularly in in vivo models. Conversely, understanding how *S. aureus* manipulates host immunity, by limiting NO production, inducing anti-apoptotic pathways, or shifting macrophage polarization, could reveal therapeutic targets to prevent persistent infections and tissue damage. Therefore, this study establishes a conceptual framework for understanding pathogen-specific host responses that could guide hypothesis-driven in vivo studies aimed at improving mastitis control strategies and reducing reliance on antimicrobials. Nonetheless, a key limitation of this study lies in the use of a macrophage cell line, which, although well-characterized and widely used, may not fully capture the functional complexity and physiological relevance of primary macrophages.

## 4. Conclusion

Our findings demonstrate that different strains of *S. aureus*, a major mastitis pathogen, and *S. chromogenes*, typically considered a minor pathogen, trigger distinct macrophage transcriptional responses from the early stages of the infection. Notably, *S. aureus* IMI strains evade immune responses by producing low nitrite despite high *NOS2* mRNA expression. The transcriptional profile induced by the commensal *S. aureus* SN strain appears to promote an M2-like profile, fostering immune tolerance and bacterial colonization. Furthermore, *S. aureus* upregulated the anti-apoptotic *Bcl2* gene, which may reflect transcriptional programs associated with cell survival pathways within macrophages. Overall, these findings underscore the role of mastitis-associated staphylococci in shaping early host transcriptional responses, which may influence infection outcomes. Therefore, the role of persistent or commensal bacteria in priming the host cell response may influence the control or progression of subsequent infections.

## Data Availability

The manuscript provides the data upon which the conclusions of the manuscript are based.
